# Immune cytopenias as a continuum in inborn errors of immunity: An in‐depth clinical and immunological exploration

**DOI:** 10.1002/iid3.420

**Published:** 2021-04-10

**Authors:** Daniele Zama, Francesca Conti, Mattia Moratti, Maria E. Cantarini, Elena Facchini, Beatrice Rivalta, Roberto Rondelli, Arcangelo Prete, Simona Ferrari, Marco Seri, Andrea Pession

**Affiliations:** ^1^ Pediatric Unit IRCCS Azienda Ospedaliero‐Universitaria di Bologna Bologna Italy

**Keywords:** autoimmune hemolytic anemia, autoimmune lymphoproliferative syndrome, autoimmune neutropenia, common variable immune deficiency, DiGeorge syndrome, immune cytopenias, immune thrombocytopenia, inborn errors of immunity

## Abstract

**Background:**

Immune thrombocytopenia (ITP), autoimmune hemolytic anemia (AIHA), and autoimmune neutropenia (AIN) are disorders characterized by immune‐mediated destruction of hematopoietic cell lineages. A link between pediatric immune cytopenias and inborn errors of immunity (IEI) was established in particular in the combined and chronic forms.

**Objective:**

Aim of this study is to provide clinical‐immunological parameters to hematologists useful for a prompt identification of children with immune cytopenias deserving a deeper immunological and genetic evaluation.

**Methods:**

We retrospectively collected 47 pediatric patients with at least one hematological disorder among which persistent/chronic ITP, AIHA, and AIN, aged 0–18 years at onset of immune cytopenias and/or immune‐dysregulation. The cohort was divided into two groups (IEI+ and IEI−), based on the presence/absence of underlying IEI diagnosis. IEI+ group, formed by 19/47 individuals, included: common variable immune deficiency (CVID; 9/19), autoimmune lymphoproliferative syndrome (ALPS; 4/19), DiGeorge syndrome (1/19), and unclassified IEI (5/19).

**Results:**

IEI prevalence among patients with ITP, AIHA, AIN, and Evans Syndrome was respectively of 42%, 64%, 36%, and 62%. In IEI+ group the extended immunophenotyping identified the presence of statistically significant (*p* < .05) specific characteristics, namely T/B lymphopenia, decrease in naїve T‐cells%, switched memory B‐cells%, plasmablasts%, and/or immunoglobulins, increase in effector/central memory T‐cells% and CD21low B‐cells%. Except for DiGeorge and three ALPS patients, only 2/9 CVID patients had a molecular diagnosis for IEI: one carrying the pathogenic variant CR2:c.826delT, the likely pathogenic variant PRF1:c.272C> and the compound heterozygous *TNFRSF13B* variants p.Ser144Ter (pathogenic) and p.Cys193Arg (variant of uncertain significance), the other one carrying the likely pathogenic monoallelic variant TNFRSF13B:p.Ile87Asn.

**Conclusion:**

The synergy between hematologists and immunologists can improve and fasten diagnosis and management of patients with immune cytopenias through a wide focused clinical/immunophenotypical characterization, which identifies children worthy of IEI‐related molecular analysis, favouring a genetic IEI diagnosis and potentially unveiling new targeted‐gene variants responsible for IEI phenotype.

## Graphical abstract

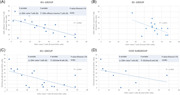

## 1 INTRODUCTION

Immune cytopenias are disorders characterized by immune‐mediated destruction of hematopoietic cell lineages. The most common form in children is immune thrombocytopenia (ITP), followed by autoimmune hemolytic anemia (AIHA) and autoimmune neutropenia (AIN).^1^ In children affected by AIHA, a link between immune cytopenias and inborn errors of immunity (IEI) was observed in 8–10% of cases.^2^ In a pediatric cohort with Evans syndrome (ES), defined as synchronous or metachronous association between AIHA and ITP, immune‐dysregulation phenomena, including IEI, were found in 70% of the patients.^3^, ^4^

IEI are congenital disorders caused by defects in genes involved in the development, function and regulation of the immune system, resulting not only in increased susceptibility to infections, but also in inflammatory, autoimmune, allergic, nonmalignant lymphoproliferative, and neoplastic manifestations.^5^, ^6^, ^7^ IEI most frequently associated with immune cytopenias are: common variable immune deficiency (CVID), autoimmune lymphoproliferative syndrome (ALPS), Wiskott‐Aldrich syndrome, and combined immunodeficiency (CID).^5^ Depending on the different forms of IEI, the pathogenic mechanisms of cytopenias identified thus far are various: humoral and cell‐mediated adaptive immunity,^8^ immune‐dysregulation in form of hemophagocytosis and splenic sequestration secondary to abnormal lymphoproliferation,^6^, ^9^ myelodysplasia,^10^ primary bone marrow failure and myelosuppression secondary to infections, malignancies and bone marrow lymphocytic infiltration.^5^ IEI‐related immune cytopenias differ from idiopathic forms on these issues: late onset, multi‐lineage involvement, chronic/relapsing course, and tendency towards treatment refractoriness.^1^ Aim of this study was to assess prevalence and clinical/immunological predictive factors of IEI in children with immune cytopenias, identifying patients worthy of IEI‐related molecular analysis.

## 2 METHODS

### 2.1 Design

This retrospective study included 47 patients since 2000–2019 suffering from immune cytopenias at Pediatric Hematology and Immunology Units of the University‐Hospital Sant'Orsola—Bologna.

Inclusion criteria were:

- Diagnosis of at least one hematological disorder among persistent/chronic ITP, AIHA, AIN;
- Age 0–18 years at initial presentation of hematological disease and/or of immune‐dysregulation.

Persistent and chronic ITP were defined as a platelet count of <100 × 10⁹/L with no known cause lasting respectively for more than 3 and 12 months.

AIHA was defined by anemia (hemoglobin < −2*SD*s) and a positive direct antiglobulin test associated to signs of hemolysis.

AIN was defined by a neutrophil count <1.5 × 10⁹/L after the 1st year of life and the detection of anti‐neutrophils antibodies using indirect flow cytometry.

ES was defined by the synchronous/metachronous presence of at least two immune cytopenias.^4^

The diagnosis of IEI met the 2019 revised criteria established by the European Society of Immunodeficiencies (ESID).^11^

We performed an extended clinical‐laboratory characterization of the cohort reported in Supporting Information table.

### 2.2 Statistical analysis

Descriptive statistics included means (95% confidence interval) as appropriate for continuous variables and frequency for categorical variables. All analyses were performed with SPSS software version 21 and Microsoft Excel version 2013. Data elaboration, based on the comparison between two groups (IEI+ and IEI−) differing in the presence/absence of a previously diagnosed IEI, aimed to:

- Assess statistical significance (*p* < .05) of a difference between the groups, through *χ* ^2^ tests for frequency and Student t‐test for mean.
- Describe the correlation (*p* < .05) between variables within a group, through Pearson correlation coefficient.

### 2.3 Laboratory investigations

- Extended lymphocyte immunophenotyping: CD3+CD4+, CD4+CD45RA+CCR7+ (naїve), CD4+CD45RA−CCR7− (effector memory), CD4+CD45RA−CCR7+ (central memory), CD3+CD8+, CD8+CD45RA+CCR7+ (naїve), CD8+CD45RA−CCR7− (effector memory), CD8+CD45RA−CCR7+ (central memory) and CD4+CD127−CCR7+CD25++ (regulatory) T‐cells (T‐reg), CD19+ (PAN−B), CD19+IgD+CD27+ (memory), CD19+IgD−CD27+ (switched memory), CD19+IgM++ CD38++ (transitional) and CD19+CD21+lCD38− (CD21low) B‐cells, CD19+IgM−+CD38++ (plasmablasts) through multiparametric flow cytometry.
- Immunoglobulin levels (IgG, IgA, IgM) through turbidimetric method.

To avoid any possible alteration linked to the immunosuppression and to maintain the setting of the immunophenotyping execution the same in both groups, immunophenotypical data were age‐referenced and gathered at least 6 months after the end of rituximab and mycophenolate mofetile (MMF), and at least 4 months after the end of steroid therapy, after the exclusion of three IEI− patients whose evaluation was executed one day after stopping and/or during a cycle of steroids. The same goes for immunoglobulin levels, which were detected at least 6 months after the end of rituximab and MMF, and at least 2 months after the end of IVIG and steroid therapy, after the exclusion of two IEI− subjects, whose measurement was made respectively during cyclic subcutaneous IgG replacement and MMF therapy. This is the reason why a more detailed B and T cell phenotyping is done in 18/19 patients in IEI+ group but only in 16/28 (57%) subjects in IEI− group. Nevertheless, this fact does not affect statistical analysis, as the number of observations is similar between the two groups (18 vs. 16) and the setting of the typization execution was the same.

### 2.4 Molecular analysis

#### 2.4.1 Targeted gene panel design

Diagnostic gene panel was developed on the Ion Torrent platform (Thermo Fisher Scientific).

The custom panel includes 46 genes involved in the pathogenesis of agammaglobulinemia, CVID, and IEI immune‐dysregulation disorders (Table 1A/1B) based on the last update of the ESID classification.^11^, ^12^

**Table 1:** IEI‐related molecular analysis through a specific IEI targeted gene panel

	Patients	Gene	Comments	cDNA	Protein	Variant classification
A (CVID)	Pt.1	CR2	Complement C3d receptor 2	c.914G>T	p.Cys305Phe	VUS
	Pt.2	CR2	Complement C3d receptor 2	c.826delT (he)	p.Cys276fs	Pathogenic
	Pt.2	PRF1	Perforin 1	c.272C>T (he)	p.Ala91Val	Likely pathogenic
	Pt.2	TCF3	Transcription factor 3	c.1331G>A (he)	p.Gly444Glu	VUS
	Pt.2	TNFRSF13B	Tumor necrosis factor receptor superfamily member 13B	c.431C>G (he)	p.Ser444Ter	Pathogenic
	Pt.2	TNFRSF13B	Tumor necrosis factor receptor superfamily member 13B	c.577T>C (he)	p.Cys193arg	VUS
	Pt.3	TNFRSF13B	Tumor necrosis factor receptor superfamily member 13B	c.260T>A (he)	p.Ile87Asn	Likely pathogenic
	Pt.4	AICDA	Activation induced cytidine deaminase	c.361G>C (he)	p.Ala121Pro	VUS
	Pt.5	CECR1	Cat eye syndrome chromosome region, candidate 1	c.927G>A (he)	p.Met309Ile	VUS
	Pt.6	LRBA	Lipopolysaccharide‐responsive beige‐like anchor protein	c.8201T>G (he)	p.Leu2734Trp	VUS
B (ALPS)	Pt.7	TNFRSF6	Tumor necrosis factor receptor superfamily member 6	c.568G>A (he)	p.Val190Met	Likely pathogenic
	Pt.8	TNFRSF6	Tumor necrosis factor receptor superfamily member 6	c.856G>T (he)	p.Gly268Ter	Pathogenic
	Pt.9	TNFRSF6	Tumor necrosis factor receptor superfamily member 6	c.580G>A (he)	p.Glu194Lys	Pathogenic
				c.673T>A (he)	p.Ser225Thr	VUS

Abbreviations: ALPS, autoimmune lymphoproliferative syndrome; CVID, common variable immune deficiency; VUS, variants of uncertain significance.

The amplicon design was expected to cover 99.97% of the 182,523 kb targeted exons with 942 amplicons. For each exon a 6 bp of padding was included.

#### 2.4.2 Ion torrent library preparation and sequencing

Ten nanograms of genomic DNA extracted from peripheral blood were used for library preparation. DNA was amplified using the two gene panel primer pools. PCR pools for each sample were combined and indexed using the Ion Xpress Barcode Adapters kit. The amplified libraries were quantified using the qPCR through the Ion Library TaqMan™ Quantitation Kit.

All samples were diluted at 10 pM, then amplicon libraries were pooled for emulsion PCR and chip loading using the Ion Chef system, according to manufacturer's instructions. The final pool of samples was sequenced with Ion GeneStudio S5 system using Ion 510 or Ion 520 Chips.

#### 2.4.3 Ion torrent bioinformatic analysis

Sequencing reads were aligned to hg19 reference genome. Variant calling was performed using Ion Torrent suite software v5.12 or Ion Reporter software v5.10. Called variants were filtered by allele frequencies, selecting variants with MAF <1% annotated with GnomAD database (https://gnomad.broadinstitute.org/). Nonsense, frameshift and canonical splicing variants were considered potentially pathogenic, while the other variants were classified according to Varsome database (https://varsome.com/). Called variants with the minimum coverage of 20× were analysed by Integrative Genome Viewer and confirmed by sanger sequencing. All regions covered <20× were reanalysed by sanger sequencing.

#### 2.4.4 DiGeorge syndrome screening

The 22q11.21 deletion was tested in 1/47 individuals according to clinical suspicion using the SALSA MLPA probemix P250 (MRC‐Holland) following the manufacturer's instructions.

## 3 RESULTS

### 3.1 Hematological presentation

**Figure 1:**
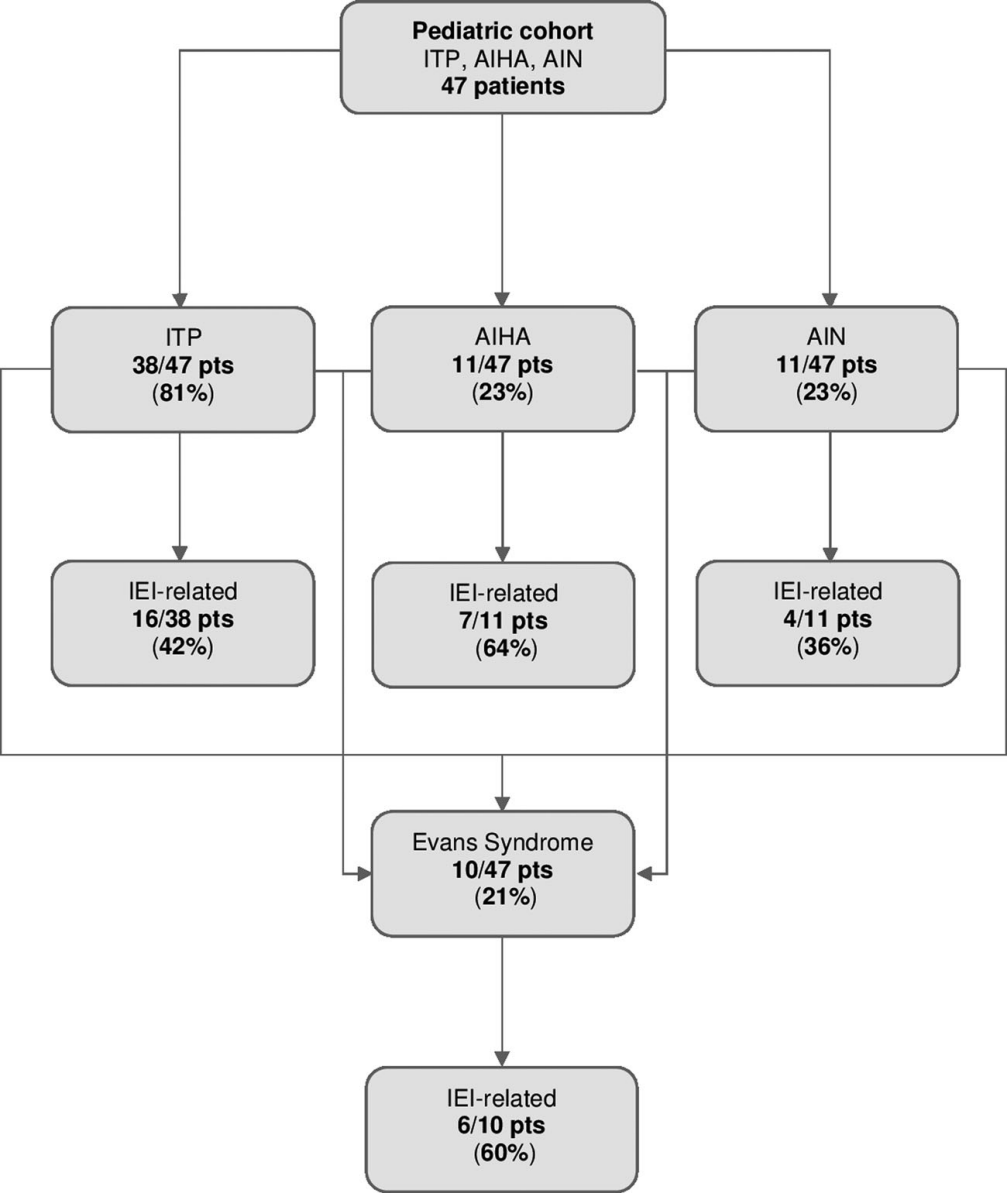
Immune cytopenias and IEI‐related forms: prevalence assessment in a pediatric cohort. AIHA, autoimmune hemolytic anemia; AIN, autoimmune neutropenia; IEI, Inborn errors of immunity; ITP, immune thrombocytopenia; pts, patients

Forty‐seven patients were included in the study. ITP was diagnosed in 38/47 subjects (81%), AIHA in 11/47 (23%), and AIN in 11/47 (23%). ES affected 10/47 individuals (21%), presenting with the following associations: ITP and AIHA in 5/10 cases (50%), ITP and AIN in 2/10 (20%), ITP, AIHA, and AIN in 3/10 (30%). IEI prevalence among patients with ITP, AIHA, AIN, and ES was respectively of 42%, 64%, 36%, and 60% (Figure 1). IEI+ group counted 19 individuals (40%), affected by CVID (9/19, 47%), ALPS (4/19, 21%), DiGeorge syndrome (DGS) (1/19, 5%) and unclassified IEI (5/19, 26%). IEI− group consisted of 28 subjects (60%). Mean age at initial presentation with immune cytopenias was not significantly higher in IEI+ group (9.7 vs. 7.9 years; range, 0.7–28.5 years; *p* = .348) and their mean follow‐up was superior in this group (11.3 vs. 4.0 years, 0.2–24.9 years; *p* < .001), probably due to a more complex clinical course of IEI‐related forms. Mean age at time of IEI diagnosis was 15.2 years (6.9–35.1 years) and mean temporal distance between immune cytopenia onset and IEI identification was 5.4 years (−7.0–18.6 years) (Table 2).

**Table 2:** General characterization of cohort, IEI+ and IEI− groups

	Total cohort (*n* = 47)	IEI+ group (*n* = 19)	IEI− group (*n* = 28)	*χ* ^2^
Categorical variables	No. (%)	No. (%)	No. (%)	*p* value
Males/females	30/17 (64/36)	15/4 (79/21)	15/13 (54/36)	.076
CVID	9 (19)	9 (47)	/a	/b
ALPS	4 (8)	4 (21)	/a	/b
DiGeorge syndrome	1 (2)	1 (5)	/a	/b
Unclassified IEI	5 (11)	5 (26)	/a	/b
Continuous variables	Mean (*SD*)	Mean (*SD*)	Mean (*SD*)	*t* test
Age at immune cytopenias onset (years)	8.6 (5.9)	9.7 (7.6)	7.9 (4.5)	.348
Age at time of IEI diagnosis (years)	15.2 (7.1)	15.2 (7.1)	/a	/b
Immune cytopenias follow‐up duration (years)	7.0 (5.8)	11.3 (6.3)	4.0 (3.0)	<.001*
Time between immune cytopenias onset and IEI identification (y)	5.4 (6.2)	5.4 (6.2)	/a	/b

Abbreviations: ALPS, autoimmune lymphoproliferative syndrome; CI, confidence interval; CVID, common variable immune deficiency; IEI, inborn errors of immunity.

* Statistically significant.

^b^ No statistical analysis conducted on the data.

^a^ Data by definition not regarding IEI− group.

Besides hematological presentation, we reported exclusively immunological parameters presenting with a significant difference between the two groups. The other variables analyzed were described in Supporting Information table.

### 3.2 Laboratory investigations

**Figure 2:**
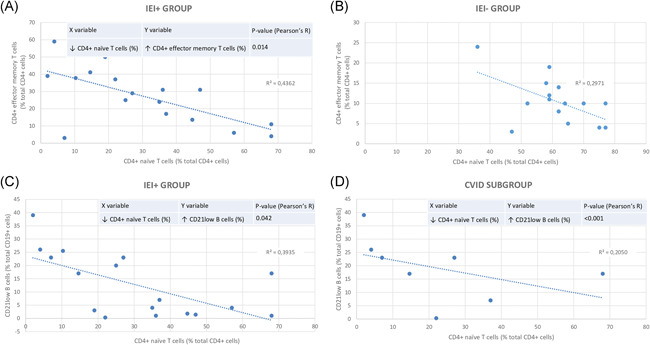
Correlations between lymphocyte subsets in IEI+/IEI− groups and CVID subgroup. (A) Scatter plot describing the correlation existing between CD4+ naïve T cells (%) (*X* axis) and CD4+ effector memory T cells (*Y* axis) in IEI+ group through Pearson's correlation coefficient. (B) Scatter plot describing the correlation existing between CD4+ naïve T cells (%) (*X* axis) and CD4+ effector memory T cells (%) (*Y* axis) in IEI− group through Pearson's correlation coefficient. (C) Scatter plot describing the correlation existing between CD4+ naïve T cells (%) (*X* axis) and CD21low B cells (%) (*Y* axis) in IEI+ group through Pearson's correlation coefficient. (D) Scatter plot describing the correlation existing between CD4+ naïve T cells (%) (*X* axis) and CD21low B cells (%) (*Y* axis) in CVID subgroup through Pearson's correlation coefficient. CVID, common variable immune deficiency; IEI, inborn errors of immunity; ↓, decrease; ↑, increase

- The altered parameters of lymphocyte typing showing a predominance in IEI+ group were the following: lymphopenia (*p* = .009), a reduction below the lower limit of normal (LLN) in CD4+ naїve T‐cells% (*p* = .001), CD8+ naїve T‐cells% (*p* = .022), T‐reg cells% (*p* = .009) and switched memory B‐cells% (*p* = .008), an increase over the upper limit of normal in CD4+ effector memory T‐cells% (*p* = .001) and CD21low B‐cells% (*p* = .003) (Table 3). There was a difference in mean lymphocyte count (*p* = .001) between IEI+ and IEI− groups. Mean CD4+ T‐cell count was inferior (*p* = .017) in IEI+ group, the same goes for mean CD8+ T‐cell count (*p* = .004). There was a difference (*p* < .001) in mean CD4+ naїve T‐cell% count between IEI+ and IEI− groups, as for CD8+ naїve T‐cell% count (*p* < .001). Mean CD4+ central memory T‐cell% count was superior (*p* = .002) in IEI+ group, the same goes for CD4+ effector memory T‐cell% count (*p* = .001) and CD8+ effector memory T‐cell% count (*p* = .002). CD19+ B‐cell count was on average lower (*p* = .002) in IEI+ group, as for CD19+ B‐cell% count (*p* = .034) and CD21low B‐cell% count (*p* = .009). There was a difference (*p* < .001) in switched memory B‐cell% count between IEI+ and IEI− groups, the same goes for plasmablast% count (*p* = .036) (Table 4). In IEI+ group, CD4+ naїve T‐cells% showed a negative correlation both with CD4+ effector memory T‐cells% (Pearson's *R* = −0.660) and CD21low B‐cells% (Pearson's *R* = −0.627). Furthermore, in CVID subgroup, we observed a negative correlation between CD4+ naїve T‐cells% and CD21low B‐cells% (Pearson's *R* = −0.453). Moreover, in IEI− group, CD4+ naїve T‐cells% showed an inverse correlation with CD4+ effector memory T‐cells% (Pearson's *R* = −0.545) (Figure 2).
- Hypogammaglobulinemia involving at least one class among IgG, IgA, IgM was more frequent in IEI+ group (89% vs. 36%, *p* = .001), the same goes for hypogammaglobulinemia involving at least two classes (68% vs. 12%, *p* < .001) (Table 3). Mean IgG count was inferior (*p* = .002) in IEI+ group, as for IgA (*p* = .010) and IgM (*p* < .001) (Table 4).

**Table 3:** Extended lymphocyte typization comparison between IEI+ and IEI− groups (qualitative variables)

		IEI+ group (*n* = 19)		IEI− group (*n* = 28)		*χ* ^2^
Variables		Obs	Prevalence (%)	Obs	Prevalence (%)	*p* value
Basic features	Leukopenia	19	9/19 (47)	28	9/28 (32)	.292
	Lymphopenia	19	8/19 (42)	28	2/28 (7)	.004
	↓ CD4+/CD8+ ratio	19	5/19 (26)	24	3/24 (12)	.248
CD4+ T‐cell subsets	↓ CD3+CD4+ T cells (%)^a^	19	4/19 (21)	24	2/24 (8)	.232
	↓ CD4+CD45RA+CCR7+ naїve T cells (%)^b^	18	11/18 (61)	16	1/16 (6)	.001*
	↑ CD4+CD45RA−CCR7− effector memory T cells (%)^b^	18	9/18 (50)	16	0/16 (0)	.001*
	↓ CD4+CD127−CCR7+CD25++ regulatory T cells (%)^b^	18	13/18 (72)	15	4/15 (27)	.009*
CD8+ T‐cell subsets	↓ CD3+CD8+ T cells (%)^a^	19	2/19 (10)	24	2/24 (8)	.806
	↓ CD8+CD45RA+CCR7+ naїve T cells (%)^c^	18	5/18 (28)	16	0/16 (0)	.022*
	↑ CD8+CD45RA+CCR7− late effector T cells (%)^c^	18	2/18 (11)	16	2/16 (12)	.900
Other cell subsets	↓ CD56+CD16+CD3− natural killer cells (%)^a^	19	2/19 (10)	24	5/24 (21)	.363
	↑ TCRαβ+CD3+CD4−CD8− double negative T cells (%)^d^	18	8/18 (44)	19	3/19 (16)	.057
CD19+ B‐cell subsets	↓ CD19+ PAN‐B cells (%)^a^	19	2/19 (10)	21	7/21 (33)	.085
	↓ CD19+IgM++CD38++ transitional B cells (%)^e^	18	5/18 (28)	16	1/16 (6)	.100
	↓ CD19+IgD+CD27+ memory B cells (%)^e^	18	4/18 (22)	16	0/16 (0)	.045*
	↓ CD19+IgD−CD27+ switched memory B cells (%)^e^	18	7/18 (39)	16	0/16 (0)	.005*
	↑ CD19+CD21+lCD38− (CD21low) B cells (%)^e^	18	8/18 (44)	16	0/16 (0)	.002*
Immunoglobulin	Hypogammaglobulinemia (at least 1 class)^f^	19	17/19 (89)	25	9/25 (36)	<.001*
levels	Hypogammaglobulinemia (at least 2 classes)^f^	19	13/19 (68)	25	3/25 (12)	<.001*

Abbreviations: IEI, Inborn errors of immunity; obs, observations; ↓, decrease; ↑, increase.

^a^ % total lymphocytes.

^b^ % total CD4+ cells.

^c^ % total CD8+ cells.

^d^ TCRαβ+CD3+ cells.

^e^ % total CD19+ cells.

^f^ Only IgG, IgA, and IgM classes were analysed.

* Statistically significant.

**Table 4:** Extended lymphocyte typization comparison between IEI+ and IEI− groups (quantitative variables)

		IEI+ group (*n* = 19)		IEI− group (*n* = 28)		*t* test
Variables		Obs	Mean (*SD*)	Obs	Mean (*SD*)	*p* value
Basic features	WBC (cell/µl)	16	5625.6 (2 419.2)	18	5664.4 (2 046.1)	.960
	Lymphocytes (cell/µl)	18	1545.0 (660.2)	22	2487.4 (951.7)	.001*
	CD3+ PAN‐T cells (%)^a^	19	76.5 (7.8)	24	73.4 (7.5)	.188
	CD4+/CD8+ ratio	19	1.4 (0.6)	24	1.6 (0.8)	.432
CD4+ T‐cell subsets	CD3+CD4+ T cells (cell/µl)	18	663.3 (358.5)	22	979.2 (429.6)	.017*
	CD3+CD4+ T cells (%)^a^	19	40.3 (10.1)	24	39.9 (6.6)	.892
	CD4+CD45RA+CCR7+ naїve T cells (%)^b^	17	30.8 (21.0)	16	61.5 (10.8)	<.001*
	CD4+CD45RA−CCR7+ central memory T cells (%)^b^	17	41.2 (15.6)	16	25.6 (9.9)	.002*
	CD4+CD45RA−CCR7− effector memory T cells (%)^b^	17	26.9 (16.3)	16	10.4 (5.6)	.001*
	CD4+CD45RA+CCR7− terminal effector memory T cells (%)^b^	17	1.0 (0.6)	16	2.1 (3.1)	.195
	CD4+CD127−CCR7+CD25++ regulatory T cells (%)^b^	17	4.3 (11.9)	15	4.1 (1.9)	.916
CD8+ T‐cell subsets	CD3+CD8+ T cells (cell/µl)	18	440.4 (160.8)	22	674.0 (301.7)	.004*
	CD3+CD8+ T cells (%)^a^	19	29.5 (6.5)	24	28.0 (8.5)	.536
	CD8+CD45RA+CCR7+ naїve T cells (%)^c^	17	30.5 (18.3)	16	51.2 (10.6)	<.001*
	CD8+CD45RA−CCR7+ central memory T cells (%)^c^	17	9.3 (6.2)	16	5.9 (3.3)	.055
	CD8+CD45RA−CCR7− effector memory T cells (%)^c^	17	35.8 (15.3)	16	21.2 (8.7)	.002*
	CD8+CD45RA+CCR7− late effector T cells (%)^c^	17	25.5 (16.6)	16	21.5 (11.1)	.424
Other cell subsets	CD56+CD16+CD3− natural killer cells (cell/µl)	18	146.1 (74.0)	22	208.9 (156.7)	.106
	CD56+CD16+CD3− natural killer cells (%)^a^	19	9.9 (4.0)	24	8.3 (4.2)	.197
	TCRαβ+CD3+CD4−CD8− double negative T cells (%)^d^	18	2.7 (1.6)	19	2.4 (3.1)	.691
	CD3+γ+δ+ (%)^a^	17	4.5 (5.9)	16	4.9 (1.5)	.661
CD19+ B‐cell subsets	CD19+ PAN‐B cells (cell/µl)	18	191.0 (150.8)	20	444.5 (284.1)	.002*
	CD19+ PAN‐B cells (%)^a^	19	12.6 (6.6)	21	17.5 (7.2)	.034*
	CD19+IgD+CD27− naïve B cells (%††)^e^	17	79.1 (9.1)	16	76.6 (7.7)	.416
	CD19+IgM++CD38++ transitional B cells (%)^e^	17	6.4 (4.3)	16	5.3 (2.5)	.371
	CD19+IgD+CD27+ memory B cells (%)^e^	17	9.8 (8.4)	16	8.0 (3.2)	.426
	CD19+IgD−CD27+ switched memory B cells (%)^e^	17	2.5 (2.9)	16	10.2 (4.2)	<.001*
	CD19+CD21+lCD38− CD21low B cells (%)^e^	17	12.6 (11.9)	16	3.9 (1.9)	.009*
	CD19+IgM−+CD38++ plasmablasts (%)^e^	17	0.7 (0.5)	16	1.6 (1.4)	.036*
Immunoglobulin levels	IgG (mg/dl)^f^	19	653.5 (369.8)	25	969.0 (264.3)	.002*
	IgA (mg/dl)^f^	19	74.5 (83.5)	25	150.8 (99.2)	.010*
	IgM (mg/dl)^f^	19	48.6 (33.0)	24	92.0 (40.1)	<.001*

Abbreviations: IEI, Inborn errors of immunity; obs, observations; WBC, white blood cells.

^a^ % total lymphocytes.

^b^ % total CD4+ cells.

^c^ % total CD8+ cells.

^d^ TCRαβ+CD3+ cells.

^e^ % total CD19+ cells.

^f^ SI conversion factor: to convert IgG/IgA/IgM to g/L, multiply values by 10^2^.

* Statistically significant.

### 3.3 Molecular analysis

3/47 patients, diagnosed as ALPS Type IA, carried pathogenic variants in the *TNFRSF6* gene, coding for FAS receptor (Table 1B). The patient affected by DiGeorge syndrome showed a 2 Mb pathogenic deletion on chromosome 22 from *CLTCL‐1* gene to *LRZTR1* gene.

Except for DiGeorge and three ALPS patients, only 2/9 CVID patients who underwent the previously described genetic analysis had a molecular diagnosis for IEI (Table 1A):

- Pt. 2 carrying the pathogenic variant CR2:c.826delT, the likely pathogenic variant PRF1:c.272C> and the compound heterozygous *TNFRSF13B* variants p.Ser144Ter (pathogenic) and p.Cys193Arg (VUS);
- Pt. 3 carrying the likely pathogenic monoallelic variant TNFRSF13B:p.Ile87Asn, as TNFRSF13B‐related CVID shows both an autosomal and recessive inheritance model.

As regards the other 7/9 subjects suffering from CVID, diagnosis met the 2019 revised clinical‐immunological ESID criteria, as CR2 (pt. 1) and LRBA (pt. 6) mutations cause CVID in an autosomal recessive manner, while CECR1 (pt. 5) and AICDA (pt. 4) mutations are so far associated with other CVID‐like syndromes. Furthermore, as in CVID more than one gene could influence the phenotype, we consider that the variants reported even in the absence of a clear genotype‐phenotype correlation could somehow impact the clinical manifestations and should be considered/mentioned as variants of uncertain significance (VUS). Moreover, further studies are needed to address these VUS as responsible for the clinical CVID phenotype. Pt. 7, 8 and 9 did not carry any variants in the gene panel analysed. No genetic testing was done in the IEI− group as patients did not meet the clinical‐immunological ESID criteria necessary to raise suspicion of Unclassified IEI.

Genetic background differences between the two groups is undoubtedly an interesting point to evaluate in the future.

## 4 DISCUSSION

This study found a strong association between immune cytopenias and IEI, demonstrating the feasibility and usefulness of an extended lymphocyte immunophenotyping to detect IEI signs in immune cytopenic pediatric patients. In this context, we identified T and B lymphopenia, a reduction in CD4+ and CD8+ naїve T‐cells%, T‐reg cells% and switched memory B‐cells%, and an increase in CD4+ effector memory T‐cells% and CD21low B‐cells% along with the presence of hypogammaglobulinemia as variables significantly associated with IEI.

Mean lymphocyte, CD4+ and CD8+ T‐cell count reached lower mean levels in IEI+ group. Lymphopenia is indeed one of the factors of *immunodeficiency related score*, a diagnostic algorithm developed for an early identification of IEI^13^; thus, it is a suggestive sign of CID,^11^ described also in individuals with CVID.^14^

A general reduction in CD4+ T‐cells probably led to a decline in T‐reg cells% under the LLN, more prevalent in IEI+ group with statistical significance (Table 3) and responsible for the impairment of self‐tolerance mechanism that frequently occurs in subjects with IEI.^15^

T‐cell phenotype in IEI+ group showed a decrease in CD4+ and CD8+ naїve T‐cells% against an expansion in CD4+ and CD8+ effector memory T‐cells%, being common features of CVID,^16^ reported also in DGS,^17^ likely due to persistent immune system stimulation by chronic/recurrent infections. Diverging from literature, we found increased CD4+ central memory T‐cells% in IEI+ group, as a potential consequence of their abnormal interaction with antigen‐presenting cells and subsequent failed differentiation into effector memory T‐cells.^18^

As concerns B‐cell compartment, it is worth highlighting the statistically significant difference between IEI+ and IEI− groups regarding the absolute (*p* = .002) and relative (*p* = .034) numbers of PAN B‐cells, which were both inferior in patients with IEI.

This suits with the fact that B‐lymphopenia is a common finding in many IEI forms, in fact it is very frequent in CVID, frequent in SCID/CID, and occasional in ALPS.^11^

B‐cell phenotype was characterized in IEI+ group by an accumulation of CD21low B‐cells% against a decrease in PAN‐B%, switched memory B‐cells% and plasmablasts%, whose lack resulted in a defective production of IgG, IgA, and IgM.

So B‐cell subsets repartition and immunoglobulin levels in IEI+ group resemble the peculiar features of CVID, probably because it is the most frequent IEI in literature^19^ as well as in our cohort (9/19, 47%) (Table 2).

In IEI+ group, CD4+ naїve T‐cells% showed a negative correlation both with CD4+ effector memory T‐cells% and CD21low B‐cells%. The negative correlation between CD4+ naїve T‐cells% and CD21low B‐cells% maintained significance in our CVID subgroup, as reported by Warnatz and Schlesier.^20^ All these associations probably reflect the role of persistent/chronic inflammation‐ and infection‐driven immune activation in the impairment of T‐cell regulation of B‐cells,^20^ favouring the development of auto‐reactive CD21low B‐cells and the differentiation from naïve to effector memory T‐cells and eventually resulting in an immunosenescent‐like phenotype typical of some IEI forms.^21^ These features were also found in patients suffering simultaneously from both CVID and ITP,^22^ characterized by an accumulation of CD21low B lymphocytes, responsible for IEI‐related autoimmune disorders,^8^ along with defects in PAN‐B cells bone marrow production and CD4+ naїve T‐cells differentiation. These findings suit also with the individual of the cohort with DGS, showing low CD4+ naїve T‐cells%, switched memory B‐cells% and IgM levels, along with an inverted CD4+/CD8+ ratio, all biomarkers configuring an immunophenotype associated with autoimmunity, lymphoproliferation and a severe clinical course, characterized in our case by invasive and recurrent infections.^23^, ^24^, ^25^

Our hypotheses concerning the above‐mentioned correlations in IEI+ group and CVID subgroup are strengthened by the fact that, in IEI− group, CD4+ naїve T‐cells% showed an inverse correlation respectively milder with CD4+ effector memory T‐cells% (Pearson's *R* = −0.545) and not significant with CD21low B‐cells% (Pearson's *R* = −0.024). Memory and transitional B‐cells% did not show a significant difference between the two groups. This result could be indicative of continuous autoantigen‐driven stimulation and defective immune‐regulatory networks, previously described in subjects with autoimmune manifestations, including ITP,^26^, ^27^ which was the most represented immune cytopenia in our two groups.

We identified a high rate of correlation between immune cytopenias and IEI, probably due to the inclusion criteria of the cohort, in which persistent/relapsing forms of immune cytopenias affected 97% of patients. Our hypothesis is supported by Hadjadj, assessing the pathogenic role and poor prognostic significance of proven and/or potential IEI‐related genes in 65% of pediatric ES cases.^28^

We found two individuals with CVID carrying *TNFRSF13B* variants. The frequency of *TNFRSF13B* gene mutations was reported to be significantly higher in CVID compared to healthy controls and TACI biallelic mutations were detected only in CVID. Patients with biallelic TACI mutations had a similar incidence of autoimmunity and lymphoproliferation compared to wild‐type TACI subjects. CVID individuals carrying monoallelic mutations had a severe clinical course with the highest prevalence of immune‐dysregulation disorders.^29^ Interestingly, the Pt.2 patient carried also three other alterations, the pathogenic variants CR2:c.826delT and PRF1:A91V and the VUS TCF3:G444E (Table 1A). Further studies are needed to validate these additional variants and to understand if they act in concert with biallelic genetic alterations in *TNFRSF13B* to give rise to complex IEI‐related phenotype.

In conclusion, IEI diagnosis in patients with immune cytopenias was significantly associated with specific clinical signs and immunophenotypical anomalies, namely T/B lymphopenia, decrease in naїve T‐cells%, switched memory B‐cells%, plasmablasts%, and immunoglobulins, increase in effector/central memory T‐cells% and CD21low B‐cells%.

This study's primary limitations result from the sample size and the retrospective nature of the review. The high diagnosis rate of IEI in such a relatively limited cohort could be related to subjects' enrollment at a tertiary care center, where immunodeficiency suspicion may be elevated.

Although the association between immune cytopenia and IEI has already been described in literature, the identification of patients with a possible IEI remains a clinical challenge, in particular for pediatric and adult hematologists.

This study highlights that a synergy between hematologists and immunologists and a deeper knowledge of the immunological features in these patients can improve and fasten these patients' diagnosis and management. Our main purpose consisted in providing immunological parameters to hematologists useful for a prompt identification of immune cytopenic children deserving a deeper immunological and genetic evaluation. First, this could favors a molecular IEI diagnosis through specific next generation sequencing panels, applied by reason of a clinical‐immunological IEI suspicion. Eventually, this could also represent a useful tool to identify new targeted‐gene VUS (Table 1), to unveil and validate through further studies their correlation with IEI phenotype, which in some cases is the expression of a complex genotypic interaction between a small number of mutant genes rather than of a single‐gene inherited Mendelian disorder.

## CONFLICT OF INTERESTS

The authors declare that there are no conflict of interests.

## AUTHOR CONTRIBUTIONS

Daniele Zama, Francesca Conti, Mattia Moratti, and Maria E. Cantarini conceptualized and designed the study, drafted the initial manuscript, and reviewed and revised the manuscript. Beatrice Rivalta designed the data collection instruments, collected data, and critically revised the manuscript. Roberto Rondelli carried out statistical analyses and critically revised the manuscript. Elena Facchini, Arcangelo Prete, and Simona Ferrari contributed to interpretation of data and critically revised the manuscript. Marco Seri carried out molecular analyses and critically revised the manuscript. Andrea Pession critically reviewed the manuscript for important intellectual content and contributed to the design of the study. All authors approved the final manuscript as submitted and agree to be accountable for all aspects of the work.

## ETHICS STATEMENT

All patients and legal tutors provided written informed consent to participate in this study. Ethics Committee of Bologna University‐Hospital Sant'Orsola approved the study protocols.

## Supporting information

Supporting information.
