# Supplementary material for: Immune cytopenias as a continuum in inborn errors of immunity: An in‐depth clinical and immunological exploration
Source: Immun Inflamm Dis. 2021 Apr 10;9(2):583–94. doi: 10.1002/iid3.420 (PMC8127541; doi:10.1002/iid3.420)
Supplement: Supplementary file 1 — Supporting information. [file IID3-9-583-s001.docx]

**Supplementary table**

Comparison of qualitative clinical, laboratory and therapeutic variables between IEI+ and IEI- groups

|  | | **IEI+ group (n=19)** | | **IEI- group (n=28)** | | **Chi2** |
| --- | --- | --- | --- | --- | --- | --- |
| **Variables** | | **Obs** | **Prevalence (%)** | **Obs** | **Prevalence (%)** | **P-value** |
| *Hematological presentation* | Immune thrombocytopenia | 19 | 16/19 (84) | 28 | 22/28 (79) | 0.630 |
|  | Autoimmune hemolytic anemia | 19 | 7/19 (37) | 28 | 4/28 (14) | 0.073 |
|  | Autoimmune neutropenia | 19 | 4/19 (21) | 28 | 7/28 (25) | 0.754 |
|  | Evans syndrome | 19 | 6/19 (32) | 28 | 4/28 (14) | 0.155 |
| *Family history* | Familial autoimmunity† | 19 | 10/19 (53) | 28 | 6/28 (21) | *0.027 |
|  | Familial malignancy | 19 | 7/19 (37) | 28 | 2/28 (7) | *0.011 |
| *Infective*  *episodes* | Infections | 19 | 16/19 (84) | 28 | 23/28 (82) | 0.853 |
|  | Invasive infections‡ | 19 | 10/19 (53) | 28 | 4/28 (14) | *0.005 |
|  | Recurrent/relapsing infections§ | 19 | 7/19 (37) | 28 | 5/28 (18) | 0.143 |
|  | Non-significant infections | 19 | 14/19 (74) | 28 | 20/28 (71) | 0.865 |
|  | Viral infections | 19 | 13/19 (68) | 28 | 13/28 (46) | 0.137 |
|  | Bacterial infections | 19 | 13/19 (68) | 28 | 15/28 (54) | 0.309 |
|  | Fungal/parasitic infections | 19 | 6/19 (32) | 28 | 2/28 (7) | *0.029 |
| *Other manifestations* | Nephro-urologic alterations | 19 | 7/19 (37) | 28 | 3/28 (11) | *0.032 |
|  | Neuropsychiatric disorders | 19 | 6/19 (32) | 28 | 4/28 (14) | 0.155 |
| *Immune-*  *dysregulation* | Immune dysregulation | 19 | 18/19 (95) | 28 | 20/28 (71) | *0.046 |
|  | Mucocutaneous immune dysregulation¶ | 19 | 13/19 (68) | 28 | 8/28 (29) | *0.007 |
|  | Pulmonary immune dysregulation†† | 19 | 7/19 (37) | 28 | 0/28 (0) | *<0.001 |
|  | Articular immune dysregulation | 19 | 1/19 (5) | 28 | 5/28 (18) | 0.204 |
|  | Allergic immune dysregulation and intolerance‡‡ | 19 | 13/19 (68) | 28 | 13/28 (46) | 0.137 |
|  | Neoplastic immune dysregulation | 19 | 1/19 (5) | 28 | 1/28 (4) | 0.778 |
|  | Lymphadenopathy/splenomegaly | 19 | 18/19 (95) | 28 | 11/28 (39) | *<0.001 |
|  | Lymphadenopathy | 19 | 10/19 (53) | 28 | 0/28 (0) | *<0.001 |
|  | Splenomegaly | 19 | 17/19 (61) | 28 | 11/28 (39) | *0.001 |
| *Laboratory*  *investigations* | Anti-platelet antibodies | 16 | 11/16 (69) | 22 | 15/22 (68) | 0.970 |
|  | Anti-neutrophil antibodies | 09 | 2/9 (22) | 09 | 6/9 (67) | 0.058 |
|  | Positive direct antiglobulin test (DAT) | 15 | 8/15 (53) | 25 | 4/25 (16) | *0.013 |
|  | Anti-nuclear antibodies (ANA) | 18 | 2/18 (11) | 27 | 2/27 (7) | 0.669 |
|  | Anti-extractable nuclear antigens antibodies (ENA) | 09 | 2/9 (22) | 12 | 1/12 (8) | 0.368 |
|  | Other auto-antibodies | 18 | 5/18 (28) | 28 | 6/28 (21) | 0.622 |
|  | ↓ C3/C4 | 18 | 3/18 (17) | 18 | 1/18 (6) | 0.289 |
|  | Poor DTP early vaccinal response§§ | 06 | 3/6 (50) | 01 | 0/1 (0) | 0.350 |
|  | Poor DTP/MMR late vaccinal response§§ | 14 | 12/14 (86) | 06 | 3/6 (50) | 0.091 |
| *Therapeutic approach* | First-line therapy | 19 | 14/19 (74) | 28 | 18/28 (64) | 0.498 |
|  | IVIG | 19 | 12/19 (63) | 28 | 17/28 (61) | 0.866 |
|  | Steroids | 19 | 12/19 (63) | 28 | 18/28 (64) | 0.937 |
|  | Second-line therapy | 19 | 7/19 (37) | 28 | 11/28 (39) | 0.866 |
|  | Mycophenolate mofetile | 19 | 4/19 (21) | 28 | 3/28 (11) | 0.329 |
|  | Sirolimus | 19 | 3/19 (16) | 28 | 1/28 (4) | 0.141 |
|  | Eltrombopag | 19 | 2/19 (10) | 28 | 9/28 (32) | 0.086 |
|  | Rituximab | 19 | 3/19 (16) | 28 | 4/28 (14) | 0.887 |
|  | Splenectomy | 19 | 0/19 (0) | 28 | 1/28 (4) | 0.405 |
|  | Other drugs (romiplostim, azathioprine) | 19 | 0/19 (0) | 28 | 2/28 (7) | 0.234 |
|  | No therapy needed | 19 | 5/19 (26) | 28 | 10/28 (36) | 0.498 |

Abbreviations: DTP, Diphtheria-Tetanus-Pertussis; IEI, Inborn errors of immunity; IVIG, intravenous immunoglobulins;

MMR, Measles-Mumps-Rubella; obs, observations. Legend: ↓, decrease; ↑, increase.

* Statistically significant.

† Familial immune-mediated disorders (endocrinological, gastrointestinal, hematological, mucocutaneous, pulmonary and rheumatological).

‡ Invasive infections (bronchopneumonia, cutaneous abscesses, osteomyelitis, diffuse and/or profound herpetic lesions). § Recurrent/relapsing infections (recurrent urinary and respiratory tract infections, mycosis/parasitosis relapsing at least one time).

¶ Mucocutaneous disorders (alopecia, aphthosis, dermatitis, granulomas, melanocytic nevi, rosacea, urticarial vasculitis and warts). †† Pulmonary disorders (asthma, bronchiectasis, granulomatous-lymphocytic interstitial lung disease, other interstitiopathies). ‡‡ Allergies (asthma, dermatitis, drug and food allergies/intolerances, rhinoconjunctivitis, Vernal keratoconjunctivitis). §§ Antibody responses to MMR and DTP vaccination detected respectively through chemiluminescent (microparticle) immunoassay and enzyme immunoassay.
